# Unraveling the Heterogeneous but Ordered Microstructure of the Nonionic Deep Eutectic Solvent Formed by Lauric Acid and *N*‑Methylacetamide

**DOI:** 10.1021/acs.jpcb.5c00282

**Published:** 2025-05-30

**Authors:** Laura X. Sepulveda-Montaño, Johan F. Galindo, Daniel G. Kuroda

**Affiliations:** † Department of Chemistry, 5779 Louisiana State University, Baton Rouge, Louisiana 70803, United States; ‡ Department of Chemistry, 98733 Universidad Nacional de Colombia sede Bogotá, Bogotá 111321, Colombia

## Abstract

The nonionic deep eutectic solvent, formed by lauric acid (LA) and *N*-methylacetamide (NMA), has been shown to have a heterogeneous molecular structure in which the LA and NMA form nonpolar and polar domains, respectively. Previous vibrational spectroscopy experiments demonstrated that the ability of the LA domains to solvate compounds was limited to long carbon chains, whereas other nonpolar molecules, such as W­(CO)_6_, were found to be solvated by both LA and NMA. These experiments were not fully compatible with the previously proposed micelle-like structure of the nonpolar domains of the LA-NMA DES. In this work, the modeling of the DES molecular structure is pursued using classical molecular dynamics simulations. The new classical model reproduces both the SAXS structural factors and the previously experimentally derived interaction map for these LA-NMA DESs. In addition, the simulation also shows that LA-NMA DESs form highly organized LA aggregates that are difficult to disorganize. Further evidence of the correct description provided by the newly derived model is obtained using a moderately polar probe: chloroform-d. Computations using the classical model have a good agreement with the solvation behavior of the probe derived from experiments, in which the location of the probe is found to be mostly within the polar domain of the DES. The computational model also demonstrates that the probe solvation is a consequence of the tightly packed LA structure, which causes nonpolar molecules to be located at the interphase of the DES nonpolar domains.

## Graphical abstract

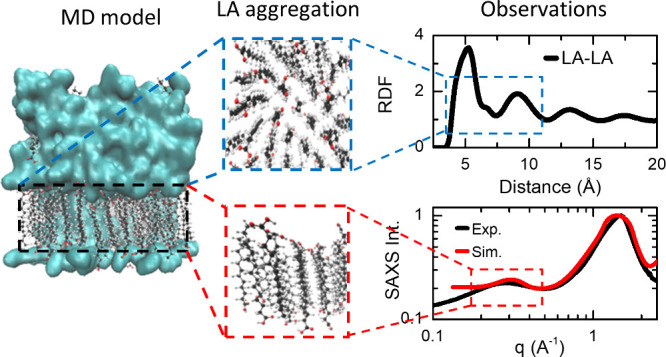

## Introduction

Deep eutectic solvents (DESs) are a class of room temperature solvents formed by combining two high melting point compounds. Due to their nonideal thermodynamic behavior, these liquids exhibit a much lower melting temperature than the individual components that form them,^1^ and are therefore commonly referred to as eutectics.^2^ While the term DES was originally applied to the mixtures of urea and quaternary ammonium salts,^3^ numerous DESs have been developed in recent years.^4–14^ The diversity of DES compositions is so extensive that they have been classified into at least five different types.^4,5,15^

DESs have a simple synthesis, usually involving mixing and gentle heating or sonication, resulting in a customizable and inexpensive family of solvents that can be prepared on demand.^16^ DESs have many desirable solvent properties, such as low vapor pressure and wide liquid range,^1,4,16,17^ making them an attractive alternative to conventional solvents, which are usually expensive and sometimes difficult to dispose of.^18–22^ Moreover, the DES binary composition allows its properties to be tuned by manipulating the composition of the mixture. Thus, there has been a growing demand for the design and use of DESs in several chemical processes, including but not limited to catalysis,^23–25^ organic and nanoparticle synthesis,^26–32^ electrochemical processes,^14,33,34^ as well as separation and extraction.^35–37^

In the past few years, research on DESs has expanded from discovery to molecular characterization. The latter is particularly important, as it opens up the possibility of tuning the DES macroscopic properties to a given application. To this end, a major focus of attention has been on the study of the DES intermolecular interactions, which include Coulombic,^38–40^ hydrogen bonds (HB),^41–43^ dipolar,^44–46^ and others, such as van der Waals (VDW).^41,47,48^ Although VDW interactions are weaker when compared to Coulombic interactions, they occur in DESs containing components with long alkyl chains.^49,50^ Nonionic DESs have only VDW and HB interactions between nonpolar and polar parts of the components, respectively, resulting in heterogeneous molecular structures.^49–52^ Similar to the case of ionic liquids,^53,54^ these molecular heterogeneous environments can solvate compounds that have a similar chemical nature to the specific regions of the DES.^55,56^ This phenomenon is not new, as it is also observed in aqueous solutions of detergents, where the surfactants can solvate nonpolar compounds by forming nonpolar domains with their long alkyl chains. Therefore, the DES heterogeneities have often been suggested to be responsible for the separation capacity of DESs in extraction processes.^55,57–59^ Furthermore, the binary composition also opens the possibility of separating the DES components by the addition of a third component, such as water.

One system that exemplifies the DESs separation capabilities is that composed of lauric acid (LA) and *N*-methylacetamide (NMA), as shown in Scheme 1. The LA-NMA DES can separate oil/water mixtures with the same gelation properties as those considered state-of-the-art.^60^ The LA-NMA mixtures present a eutectic composition at a molar ratio of 1:4 LA:NMA with a melting point of ∼6 °C, but the 1:2 and 1:6 LA:NMA molar ratios also produce liquid mixtures at room temperature, thereby expanding their applicability.^61^ Structural characterization of the LA-NMA DESs by SAXS revealed nanoscopic heterogeneities, characterized by a prepeak at 0.27 Å^–1^ in the reciprocal space, or equivalent to 23 Å in real space. Moreover, the SAXS prepeak intensity has a linear dependence on the concentration of LA, indicating that the heterogeneity arises from the presence of LA.^61^ Linear and nonlinear IR spectroscopies have also showed that the LA-NMA DESs exhibit HB interactions between the components (i.e., NMA-NMA, LA-LA, NMA-LA, and LA-NMA), where both molecules act as hydrogen bond donors and acceptors.^61^ The polar nature of NMA and nonpolar characteristics of LA, and the increase in intensity of the prepeak in SAXS with the addition of LA, have been previously used to postulate that the heterogeneous domains are formed by the aggregation of LA molecules in a continuous liquid matrix of NMA molecules.^61^ In particular, it has been proposed that the LA aggregates have a micelle-like structure, which is stabilized by the interactions among nonpolar LA alkyl chains and among LA polar heads and NMA at the interphase.^61^ The presence of well-defined polarity domains not only explains the separation power of the DES for oil/water mixtures but also brings the possibility of using the LA-NMA DES as polarity-based separation media.

**Scheme 1:**
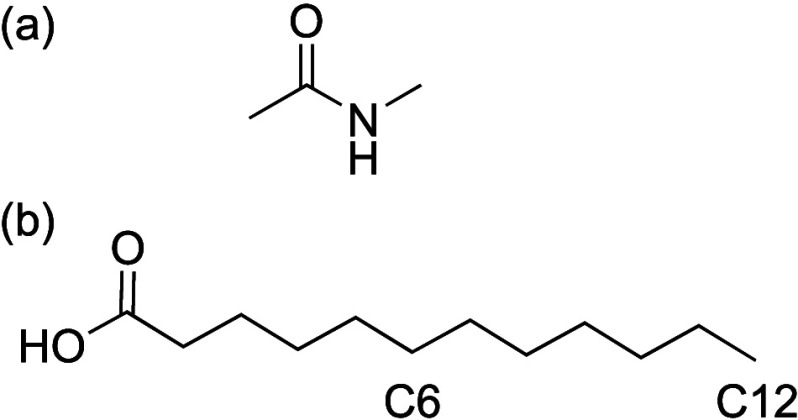
Molecular Structure of (a) *N*-Methylacetamide (NMA) and (b) Lauric Acid (LA)^a^

a C6 and C12 denote the numbering of the carbon atoms in LA.

The ability of the LA-NMA DES to accommodate different subtracts within its molecularly heterogeneous structure was previously tested using three molecular solutes.^62^ The selected molecular solutes were the polar and ionic tetrabutylammonium thiocyanate (TBASCN), the polar and nonionic benzyl thiocyanate (BSCN), and the nonpolar and nonionic tungsten hexacarbonyl (W­(CO)_6_).^62^ While the TBASCN and BSCN were found in their expected regions, NMA domain and interphase, respectively, the metal carbonyl showed an inconsistent behavior with its nonpolar character and the presence of nonpolar LA domains in these DESs.^62^ These results open new questions about the structure of the LA aggregates in the DES.

In this work, computer simulations are used to shed light on the molecular structure of the LA-NMA DES, with particular emphasis on characterizing the atomistic structure of the nonpolar domains. For this purpose, classical molecular dynamics (cMD) simulations are used to build a molecular model of the DES that can explain its structure, as well as the behavior of the different solutes. The choice of cMD as a method to sample the atomistic structure of the LA-NMA DES is driven by its computational efficiency and its ability to provide a reasonably accurate molecular representation of the structure and dynamics of the system.^63^ Special attention is given to validating the cMD model by corroborating the experimental observables. For example, a good cMD model should produce SAXS structure factors (*S*(*q*)) that are consistent with those derived from the experiments, and also should capture the different chemical interactions for each moiety as observed from IR spectroscopy.^64^

The cMD model is further validated by studying a new molecular probe (i.e., chloroform-d). The selection of chloroform-d (CLFd) is based on its moderately polar character,^65,66^ its built-in vibrational probe (i.e., C–D group),^67–69^ and its simple cMD force field representation.^70^ The moderately polar character of chloroform (39.1 on the ET scale,^65,66^ compared to 52.1 of NMA) and its lack of strong directional interactions make it suitable for testing the residence of the probe in both the polar (NMA) and nonpolar (LA) domains of the LA-NMA DES. Complementarily, the residence of the probe in the different DES regions is obtained experimentally using the C–D stretch, which is located in the 2200–2300 cm^–1^ region and has shown excellent solvatochromism properties.^69^ In order to connect the spectroscopic results to the chemical environment observed by CLFd in the cMD simulations, the recently developed IFM method is used.^64^ The IFM method computes the instantaneous vibrational frequencies of CLFd from the cMD simulations, enabling the calculation of vibrational observables, such as correlation times and central frequencies of the molecules, regardless of the solvent complexity.^64^

## Methods

For simplicity, the LA:NMA mixtures with 1:4 and 1:6 LA:NMA molar compositions are referred to as DES4 and DES6, respectively, in the remainder of the manuscript. Although the LA:NMA 1:2 molar composition is also is also a liquid at room temperature,^61^ it has not been taken into consideration in the present manuscript. The experimental procedures used to determine the experimental central frequencies can be found in the Supporting Information.

### Computational Methods

#### DESs

The study of the DES systems was performed with cMD simulations by building an initial cubic box with a side length of 50 Å using PACKMOL,^71^ with a 1:4 or 1:6 molar ratio composition for the LA and NMA molecules, respectively. Following the interpretation of previously reported experimental results,^61^ the NMA and LA molecules were arranged to form a bilayer system in which the LA tails interact with each other while their polar heads pointed outward toward the NMA domain. Note that in randomly packed 100 Å cubic boxes, the NMA and LA remained mostly mixed after 100 ns of simulation time due to the time scale of the nucleation process.^72^ Hence, these simulation results were not included as they do not agree with experimental results, such as those from SAXS.^61^ The parametrization for all simulations was made using the Generalized Amber Force Field2 (GAFF2).^70^ The system initially went through minimization, followed by a heating step (0–300 K), and a production run for 200 ns in the *NPT* ensemble. In each stage, the time step was set to 2 fs. From the first cMD simulation run, the appropriateness of the representation of the cMD model was evaluated by calculating the density of the system. A second cMD simulation run was performed for a 100 Å cubic box built in PACKMOL by replicating the original equilibrated box eight times. A new set of minimization, heating, and production followed with the previous parameters, except for the production, which in this case was run for 100 ns. In the case of DES6, a simulation box of 100 Å was run for 40 ns. The *S*(*q*) was calculated using the TRAVIS package for 100 frames from 10 ns of production,^73^ with the Lorch windowing correction applied to correct for the finite box size. The evolution and equilibration of the DES structure were evaluated using the heterogeneity order parameter *h*, since it provides an estimate of the degree of aggregation in a system and its evolution.^74^ The h parameter provides enough evidence for the equilibration of the DES4 and DES6 structures (see Supporting Information). Short length scale interactions were assessed by evaluating the radial distribution functions over the 200 ns of the production run, since the latter are not affected by the value of h (see Supporting Information), as this order parameter shows how heterogeneous the system is.

#### Molecular Probe

The behavior of CLFd was investigated in the DES4 system. In this case, CLFd was inserted in an optimized 50 Å cubic box at a concentration of 100 mM, using the GAFF2 force field for the cMD parametrization of the molecule. The chosen system consisted of both molecular solvents (dimethyl sulfoxide (DMSO), tetrahydrofuran (THF), and hexane (HEX)) as well as the DES4. All cMD simulations for the probe in different solvents were run with the same parameters as those for pure DES4, but with a production run of 200 ns.

#### MD-IFM for the Molecular Probe

In order to achieve the sampling necessary to obtain the solvatochromism of the molecular probe in different chemical environments, an additional 250 ps with a time step of 0.5 fs of production time was calculated for the systems mentioned above. In particular, for the CLF in DES4, two molecules of CLF were considered, one embedded in the LA domain and a second embedded in the NMA domain, while the rest of the molecular systems were evaluated from a single CLFd molecule embedded in each solvent. Finally, the MD-IFM method was applied as previously reported.^64^ Briefly, solvation shells were trimmed from each frame of the dynamics containing the CLF molecule and as many solvent molecules as necessary to account for the first two solvation shells. The instantaneous frequencies of the probe in this solvent cluster were later evaluated using the semiempirical method GFN2-xTB.^75^ Finally, the central frequencies for the C–D stretch mode were calculated by using the intensity-weighted average of the frequencies generated in each step of the trajectory in each solvent.

## Results

### Structure of the Pure LA-NMA DESs

The atomistic arrangements of the components in pure DES4 and DES6 were first derived from the cMD simulations using the radial distribution functions (RDFs). For this purpose, the carbonyl groups of NMA and LA, as well as some of the alkyl groups of LA, were selected to investigate the different molecular interactions occurring between components of the LA-NMA DESs. The RDFs for the selected moieties are presented in Figure 1, where in general, a very small difference is observed when comparing the different moieties in the two DESs.

**Figure 1:**
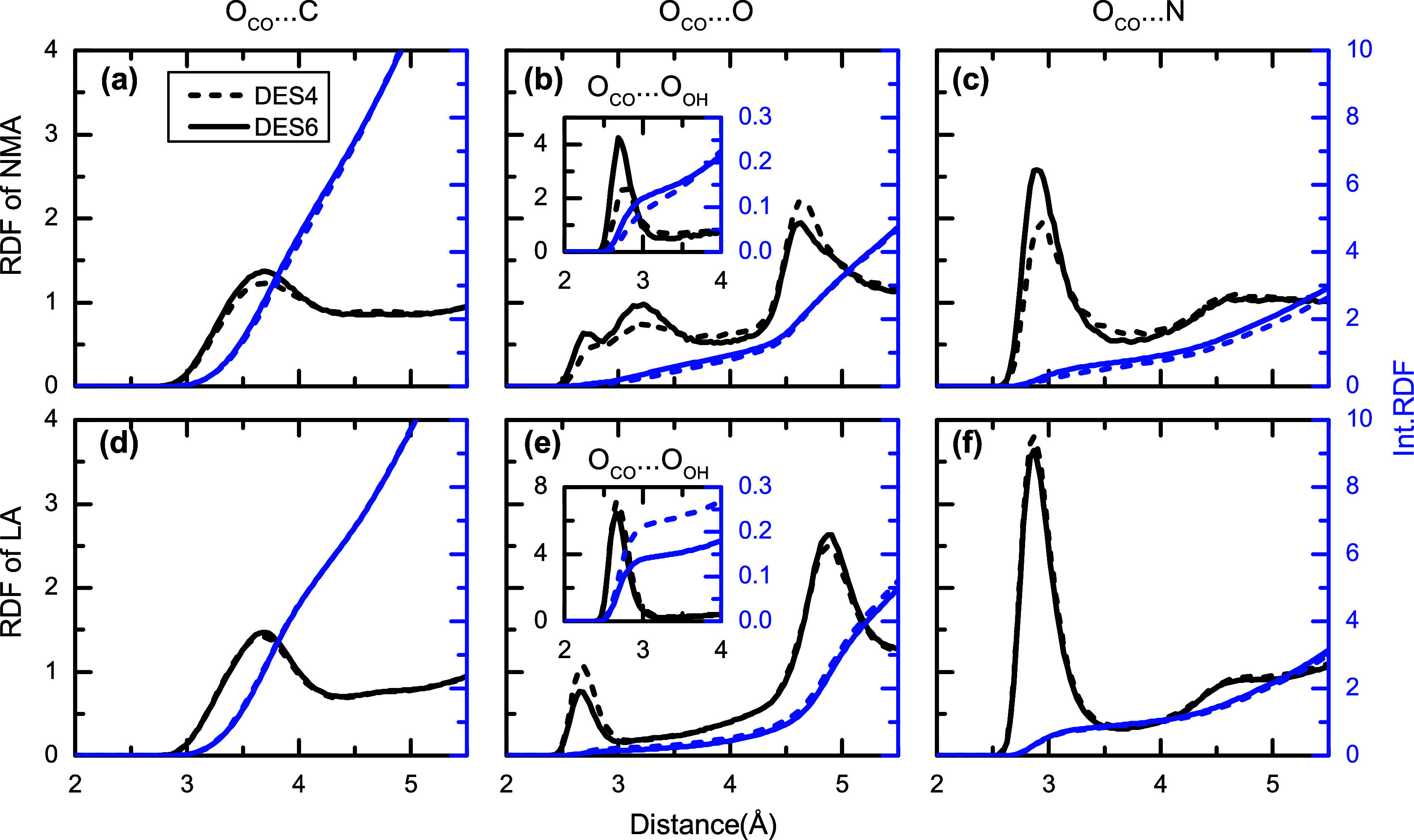
Radial distribution functions (RDF, black left axes) and integrated RDF (blue right axes) for the different moieties of NMA and LA versus a specific atom, as specified on the top of the panels. Panels (a), (b), and (c) correspond to the carbonyl oxygen atom of NMA and carbon, oxygen and nitrogen atoms of the DESs. Panels (d), (e), and (f) correspond to the carbonyl oxygen atom of LA and carbon, oxygen and nitrogen atoms of the DESs. Insets in panel (b) and (e) showcase the specific case of oxygen atom of the hydroxyl group. Dashed and solid lines correspond to DES4 and DES6.

The RDFs of the carbonyl group of NMA show that the group is solvated by an average of 4.5 molecules at distances of less than 4 Å, as observed by the RDF of the NMA carbonyl oxygen versus any carbon atom , which readily accounts for all possible moieties as all molecules have carbon atom backbones (Figure 1). When considering the coordination number of NMA molecules having a nitrogen or oxygen atom at distances less than 3.55 Å (∼0.86 and ∼0.34, respectively), only 27% of these 4.5 molecules form HBs,^76^ using the distance criteria.^77^ The integrated RDF also shows that the NMA carbonyl group forms slightly more than one HB in either DES. In addition, the probability of NMA carbonyls forming an HB with the hydroxyl group of LA is ∼27% and the remainder corresponds to NMA-NMA HBs. Interestingly, the RDFs also show that NMA molecules are solvated by ∼3 alkyl moieties from either NMA or LA molecules.

The molecular environment of LA is investigated separately for its two main parts: the polar headgroup and the hydrophobic tail. The LA headgroup, as represented by the carbonyl moiety, is surrounded by almost the same number of molecules as NMA (i.e., 4.3) at distances shorter than 4 Å, but only one of these molecules forms an HB, irrespective of the amide concentration. These HBs involve the LA carbonyl and either the -OH group of other LA molecules or the -NH group of NMA, but the highest probability is found for LA-NMA interactions, which is 61%.

A more qualitative understanding of the first solvation shell structure and HB interactions of the LA-NMA systems can be obtained by evaluating the spatial distribution functions (SDF) of the NMA and LA molecules embedded in the polar and nonpolar domains, respectively (Figure 2). Based on the RDF results (Figure 1), which show minimal changes between the structures of DES4 and DES6, the SDF analysis was performed only on DES4. In the case of NMA, the HB interactions are dominated by NMA-NMA HBs, as is observed by the large interactions of the NMA carbonyl with the NH groups of other NMA molecules. Similarly, the NMA -NH group interacts with the carbonyl group of other NMA molecules via the oxygen atom. In contrast, the HB behavior (acceptor and donor) of NMA was not observed with either the carbonyl or hydroxyl groups of LA, indicating the segregation of the latter. In the case of LA, the first solvation shell was evaluated by the interactions of the carbonyl oxygen atom, which is dominated by HB with the NH group of NMA molecules at the interphase. This effect is readily observed in the SDFs, since LA carbonyls interact with NMA molecules above the interphase of the LA domain, while lateral interactions are dominated exclusively by HBs with hydroxyl groups of other LA molecules. This interaction map is consistent with the lateral aggregation of LA molecules, which only allows interaction with NMA molecules from above.

**Figure 2:**
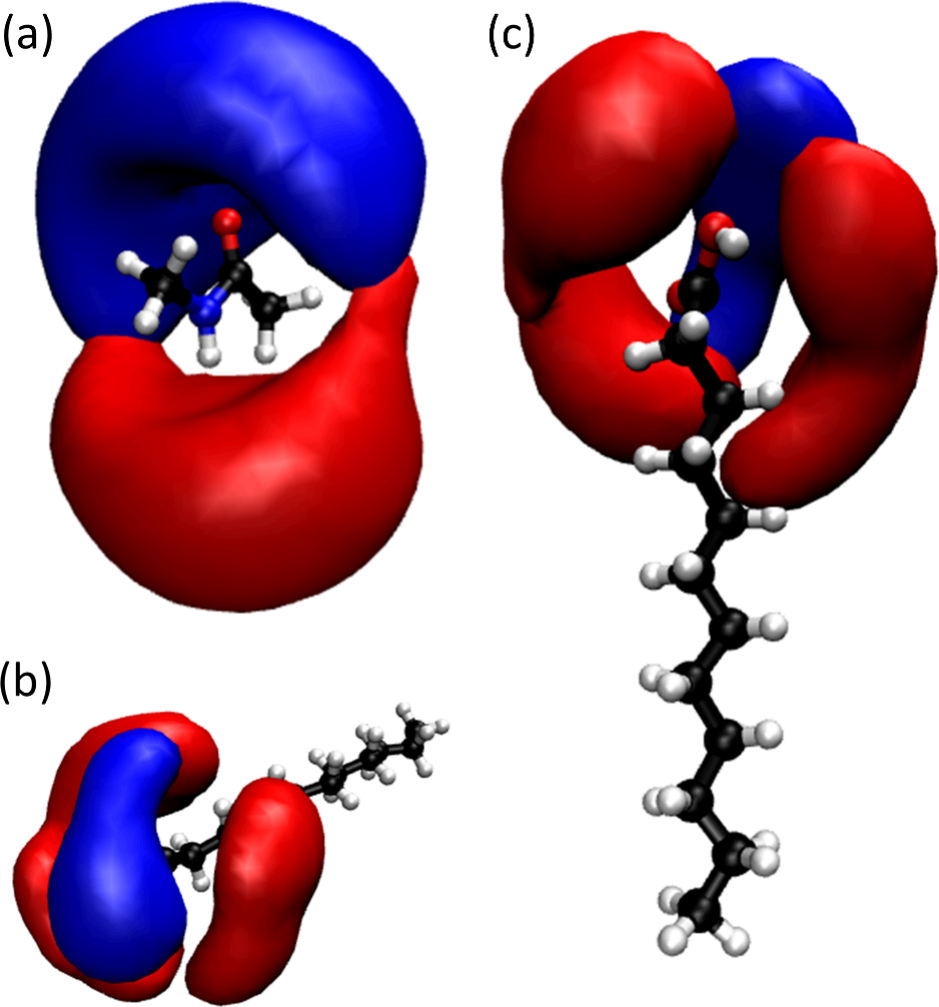
Spatial distribution functions (SDFs) plot for HB interactions in DES4. Panel (a) corresponds to the NMA interactions between the NMA O and the NH of other NMA molecules (blue SDF) and between the NH group and O of other NMA molecules (red SDF). Panels (b) and (c) to the LA interactions between the carboxylic O and the NH group of NMA (blue SDF) and the OH group of other LA molecules (red SDF) from an angular and vertical perspective, respectively

The alkyl groups of the LA tail are used to describe the hydrophobic interactions or equivalent VDW interactions that occur on the DES. In order to better discern between the different parts of the LA tail, the alkyl groups in the middle (C6 in Scheme 1) and at the end (C12 in Scheme 1) are studied. In both cases, the RDFs of C6 and C12 display an oscillatory behavior with a periodicity of ∼4 Å (Figure 3), but in the case of the tail end, it is less pronounced than that of the midsection. For the midsection (C6), the integrated RDF shows that each LA molecule is surrounded by an average of ∼3.4 LA molecules in DES4 and ∼2.7 in DES6. In contrast, the terminal alkyl group (C12) is found to be surrounded by fewer alkyl groups (∼2.4 in DES4 and ∼2.0 in DES6) than those of the midsection.

**Figure 3:**
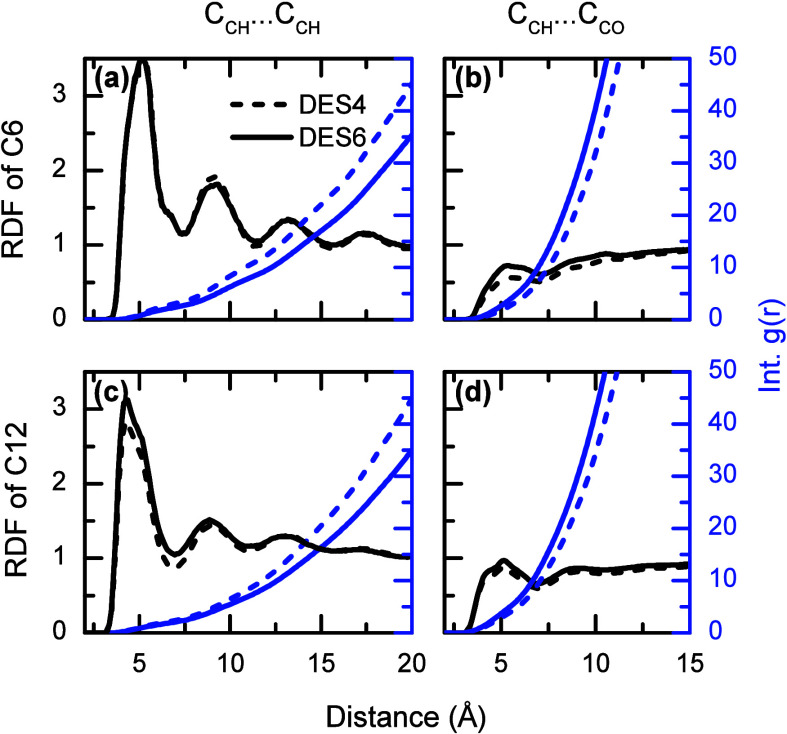
Radial distribution functions (RDF, black left axes) and integrated RDF (blue right axes) for the moieties of the LA tail. Panels (a) and (b) correspond to those of the central carbon of LA (C6) and the central carbon of other LA molecules and the carbonyl carbon of NMA, respectively. Panels (c) and (d) correspond to those of the methyl carbon of LA (C12) and the methyl carbon of other LA molecules and the carbonyl carbon of NMA, respectively. Dashed and solid lines correspond to DES4 and DES6.

The molecular structures of the modeled DESs are also evaluated by calculating the SAXS profile (*S*(*q*)) for DES4 and DES6 (Figure 4). The computed structural factors show that both DESs have a peak at ∼1.5 Å^–1^ and a prepeak below 0.5 Å^–1^. While the main peak indicates the presence of noncovalent interactions in both DESs, the small peak at low Q showcases the presence of large spatial molecular segregation occurring in DES4 and DES6.^61^

**Figure 4:**
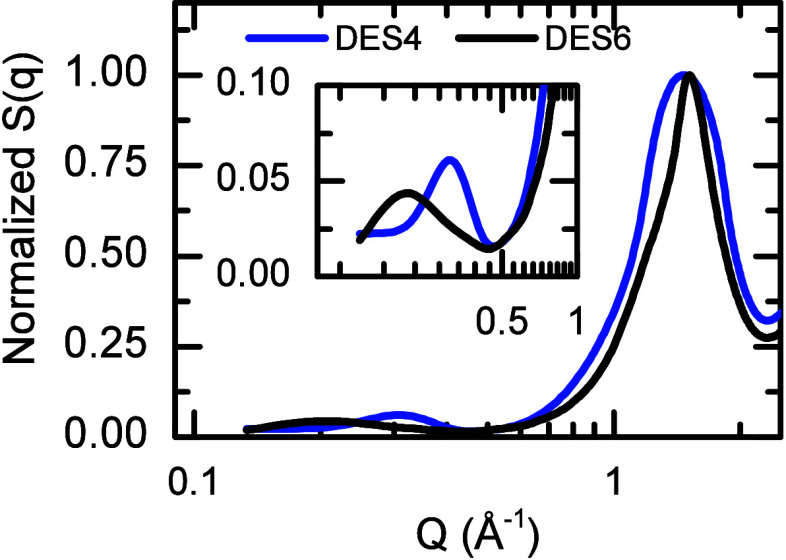
Calculated SAXS structure factor *S*(*q*) for DES4 (blue) and DES6 (black). The inset shows a close-up of the *S*(*q*) in the region of the prepeak (*Q* < 0.5 Å^–1^).

From a dynamics point of view, the diffusion of the system was investigated by assessing the mean square displacement of the different molecular components. The diffusion coefficients (*D*) for the LA and NMA molecules show interesting dynamics. The self-diffusion coefficient of NMA does not vary significantly between DES4 and DES6 (*D*_NMA_ of 0.065 and 0.078 Å^2^/ps), indicating a slight change in the NMA dynamics even when DES6 has 33.3% more NMA as compared to DES4. The time scale of the NMA diffusion is consistent with NMA molecules having bulk dynamics, which is similar in both DESs and agrees with the segregation of the different components. As in the case of LA, the changes in the diffusivity from DES4 to DES6 are substantial (*D*_LA_ = 0.012 to 0.0226 Å^2^/ps), showcasing the faster LA diffusion dynamics, or equivalently, more mobile LA domains.

### Molecular Probe in DES4

The solvation behavior of DES4 is also investigated using the moderately polar probe (chloroform-d, CLFd) and cMD simulation. The RDF for the carbon atom of CLFd (Figure 5a) and the carbon atoms of the DES components show broad peaks at a distance less than 7.5 Å for both DES components (Figure 5a). In addition, the RDF for the carbon atom of CLFd and the oxygen atoms of the carbonyl group of LA and NMA shows a peak at ∼4.5 Å or lower (Supporting Information). The integrated RDF of the central atom of CLFd and the carbonyl oxygen atom of LA is 0.2, in contrast to the value of 0.9 found for the same oxygen atom of NMA (see Supporting Information). Further characterization of the chemical environment experienced by CLFd is obtained from the solvation number evaluated in each frame of the trajectory with a cutoff of 6.0 Å from the central atom of CLFd in DES4 (Figure 5 and Supporting Information). The distribution of solvation numbers shows that the most likely solvation shell contains ∼11 solvent molecules. However, the probe is likely to be solvated by 9 to 13 DES molecules, with a 77% probability that the molecule is an amide.

**Figure 5:**
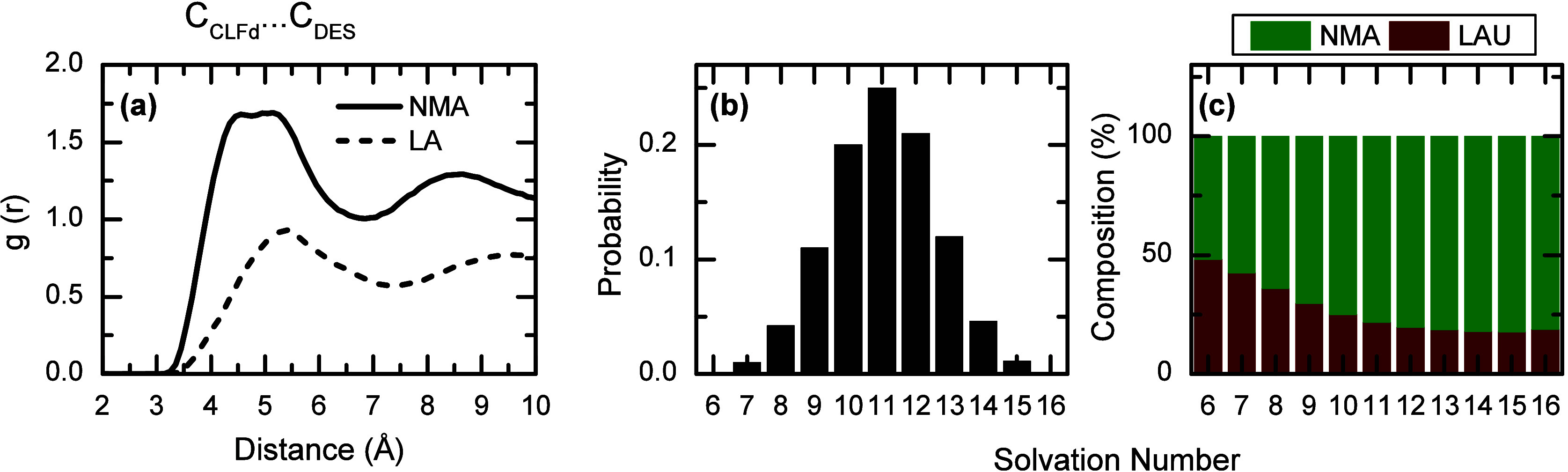
Characterization of the solvation structure of the CLFd probe in DES4. Panel (a) corresponds to the RDF from the carbon of CLFd with respect to all the carbon atoms of NMA (solid black line) and all the carbons of LA (dashed black line). Panel (b) presents the probability of finding solvation shells with different solvation numbers for CLFd in both components of DES4 taken with a cut off of 6.0 Å. Panel (c) presents the weighed probability of finding NMA (green) or LA (brown) in the first solvation shell of CLFd at a cut off of 6.0 Å.

## Discussion

### Pure DES Model

A molecular map of the interactions is obtained from the RDFs and SDFs derived from the cMD simulations (Figures 1 and 2). The RDF for the oxygen atoms of the carbonyl group of either LA or NMA shows a peak at distances shorter than 4 Å, which corresponds to the moieties of the molecules at the HB distance of the carbonyl group. While these RDFs reveal that both LA and NMA form HBs as acceptors, it is also inferred that NMA forms a single HB with other NMA molecules. On the contrary, the LA headgroup does not form a significant number of HBs and remains mostly free. Moreover, the probability of having LA–LA headgroup interactions does not change significantly in DES4 and DES6, although the relative molar concentration of LA is much lower in the latter case. This result is consistent with the previously proposed molecular structure of the DES, in which most LA molecules are found forming large aggregates (Figure 6). The aggregation of LA molecules in the DES is also supported by the RDFs of the alkyl groups both in the midsection and at the end of the alkyl tail (Figure 3). In these cases, the oscillatory RDFs demonstrate that the alkyl groups of the LA tail not only tend to interact with other LA tails, but also have a significant degree of organization. The formation of large LA aggregates agrees with the similar probability of finding LA molecules forming an HB with an NMA molecule in DES4 and DES6, since most LA molecules have their head groups in the interphase of the aggregates and are either interacting with NMA molecules (Figure 2) or free. In the case of NMA, the RDFs (Figures 1 and 2) also show that the amide has a single HB interaction with other NMA molecules, regardless of the LA concentration in the DES. The behavior of NMA in the DESs is similar to that previously described for liquid NMA.^78^ Overall, the interaction model derived from the cMD simulations is consistent with the model proposed from FTIR and SAXS experiments, in which NMA and LA molecules interact with both NMA and LA molecules, as a consequence of the formation of LA aggregates.^61^ Hence, the RDFs and SDFs demonstrate that the cMD model provides a semiquantitative representation of the short-range inter- and intramolecular interactions occurring in DES4 and DES6.

**Figure 6:**
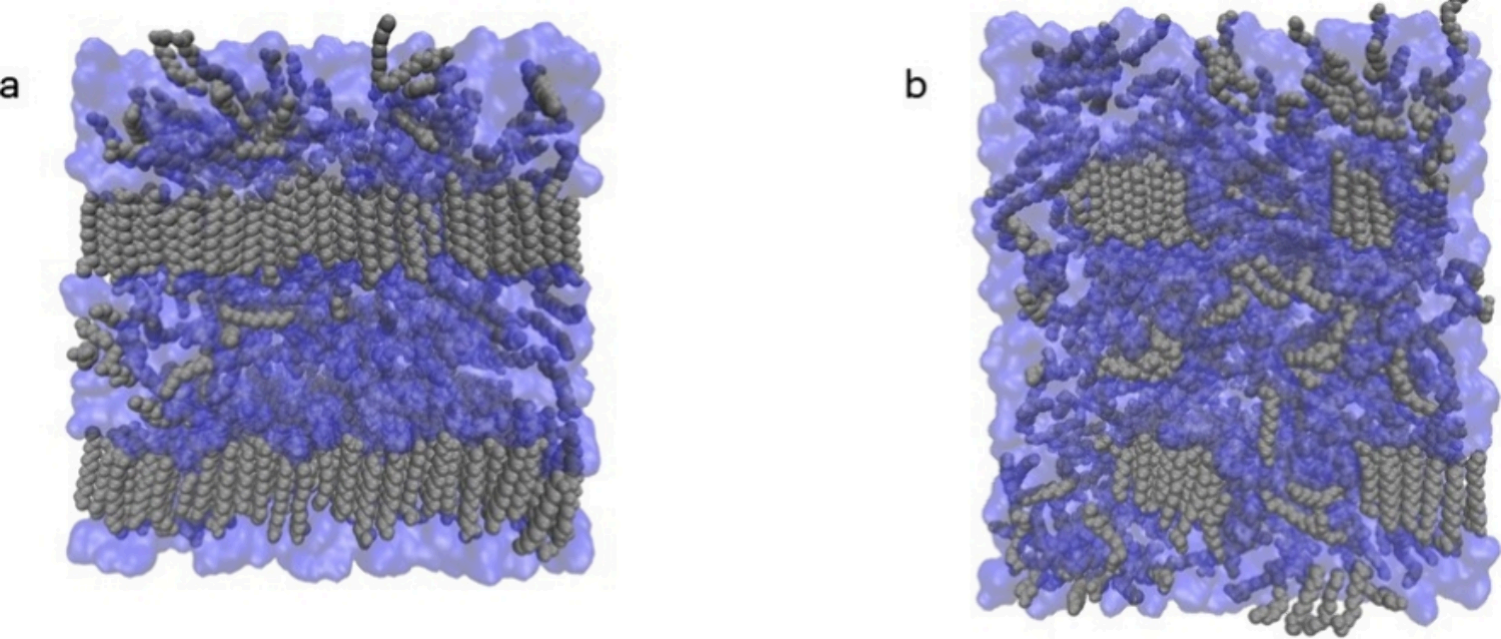
Organization of the NMA (blue) and LA (gray) domains in (a) DES4 and (b) DES6. The LA alkyl chain hydrogen atoms have been omitted for clarity.

The presence of LA domains, previously observed via SAXS, is also captured by the cMD model, as shown by the reasonable agreement between experimental and computational *S*(*q*) (Figure 4 and ref 61). Specifically, the LA aggregates in the DESs give rise to a prepeak in the *S*(*q*) seen in the experimental data at *Q* = 0.27 Å^–1^, or equivalent to 23.3 Å in real space. Note that the location of the prepeak is not well characterized in the MD simulation due to the relatively small size of the system studied (see Methods). The cMD simulations also explain why the LA aggregates are predominant in the SAXS structure factors in both DESs, as they present a highly ordered and packed structure. The order of the aggregate arises from LA tail–tail interactions, which are evidence by the oscillatory RDFs of the alkyl groups located in the middle (C6) and at the end (C12) of the LA tails (Figure 3). This periodicity of the peaks (∼4 Å) is commonly seen in very stable and organized systems.^79^

Evidence of the highly organized structure of the LA domains is also derived from geometric descriptors of the LA molecules. To this end, the degree of organization of the LA domains is further evaluated by computing the dihedral angles for the LA tail carbon backbone, since the presence of large amounts of *gauche* conformation indicates more disorganized domains.^80^ As in the RDFs, the dihedrals were evaluated in the midsection (C5–C8) and at the end (C9–C12) of the tail. The distributions (Figure 7) show two conformations of the LA backbone: the *anti* (∼±180°) and the *gauche* (∼±60°) for both sections of the tail. For the midsection, the *anti* conformation accounts for ∼90% of the distribution in both DES4 and DES6, while the rest corresponds to the gauche conformation. A similar distribution with slightly lower proportions (∼87%) is observed for the *anti* conformation at the end of the tail. The high percentage of the *anti* conformation in both parts of the LA tail indicates hindered rotation around the C–C bond of the alkyl chains in the LA molecules or, equivalently, a structure of LA in which the alkyl chains are mostly extended. A similar observation is derived from the distribution of distances between C1 and C12 in the LA molecules (Supporting Information). In this case, most LA molecules (91% for DES4 and 87% for DES6) have an extended conformation with a head-to-tail distance between 12 and 14 Å, in excellent agreement with the LA structure derived from the dihedral angles. The large number of *anti* conformations in the LA tails demonstrates that the LA aggregates are highly organized.

**Figure 7:**
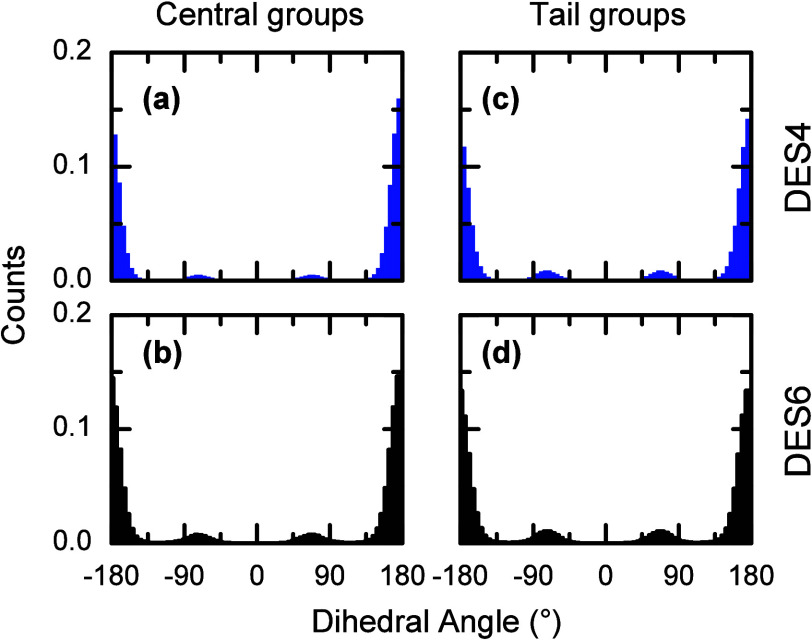
Normalized dihedral angle distributions for the alkyl chains of LA within the DESs. (a, b) Mid and tail sections of DES4, respectively, and (c, d) mid and tail sections in DES6, respectively.

Thus far, the domains seen in the LA-NMA DESs are found to be largely governed by LA-LA tail interactions as well as NMA-NMA HBs and, to a much lesser extent, by NMA-LA interactions. Notably, the interphase of the LA aggregates is composed of the LA head groups and the terminal alkyl groups of the LA tails. Interestingly, the alternating arrangement of the LA molecules suggests that the stabilization of the interphase arises from the aggregation of the LA tails and, to a lower degree, from the formation of HB bonds with NMA. Overall, the cMD model shows that the LA-NMA DESs have segregated, but highly organized LA domains, in which the LA molecules are fully extended in an interdigitate arrangement (Figure 6), with both the polar heads and tail ends pointing toward the NMA domain. This type of arrangement in the nonpolar tails has been previously observed in hydrophobic DESs formed from fatty acid mixtures.^81,82^ Consequently, the LA-NMA DES more closely resembles nanodomains of crystalline-like LA molecules suspended in liquid NMA than an ideal solution or even a micelle-like structure as previously proposed.^61^

The cMD model presented above explains the anomalous behavior of the metal carbonyl (W­(CO)_6_) in the LA-NMA DESs.^62^ In particular, it is observed that the metal complex cannot be solvated exclusively by the nonpolar tails of LA as its bulkiness (O–O distance of ∼6.5 Å, see the Supporting Information) requires a large disorganization of the tightly packed LA aggregate. However, it is likely that the solvation energy is not large enough to overcome the LA domain organization. Hence, when W­(CO)_6_ is dissolved in DES4 or DES6, it resides in the interphase of the LA domains to minimize its hydrophobic interactions, but it is forced to interact with NMA.

### Molecular Probe in DES4

The modeling and understanding of the LA-NMA DES deduced from the simulations are further tested with the new molecular probe. In the case of CLFd in DES4, the RDFs for the probe show that there are small differences between the molecular arrangements in terms of the location of the carbon atoms of both LA and NMA, indicating that CLFd is solvated by both components of the DES. This picture of the solvation shell of CLFd is consistent with that directly obtained by computing its instantaneous solvation number (Figure 5), where an average of 9 to 13 solvent molecules (NMA and/or LA) solvate CLFd. It is also observed that the composition of the solvation shell varies from having 25% of LA molecules at a solvation number of 9 to 20% when the number of solvating molecules is 13 (Figure 5). This change in the solvation shell number and composition of the probe demonstrates the large number of different solvation environments experienced by the probe in the DES. However, the average value of 23% LA molecules is very close to the statistical composition of the DES4, indicating that there is an almost negligible preferential solvation of the probe by LA molecules.

From the perspective of the polar and nonpolar domains, the cMD simulations show that the probe is mainly located in mixed or pure NMA domains (94%, Figure 8) and spends the rest of the time in the nonpolar LA domain. However, the probe is not always found in disorganized mixed domains, as in some instances it is found within LA channels capped by NMA molecules (Figure 8). In addition, the cMD simulations reveal that CLFd forms a weak HB with both DES components, as shown by the RDFs of the probe with the carbonyl groups (Supporting Information). As in the case of the solvation shell composition, the probability of CLFd forming an HB is similar to that dictated by the composition (i.e., 81% for NMA and 19% for LA). This weak HB between the probe and LA and NMA arises from the interaction between the C–D group and the carbonyl groups, as previously demonstrated.^83^ The overall cMD simulation results indicate that the CLFd is likely to remain in domains with a significant amount of NMA corresponding to either the interphase or the polar domain.

**Figure 8:**
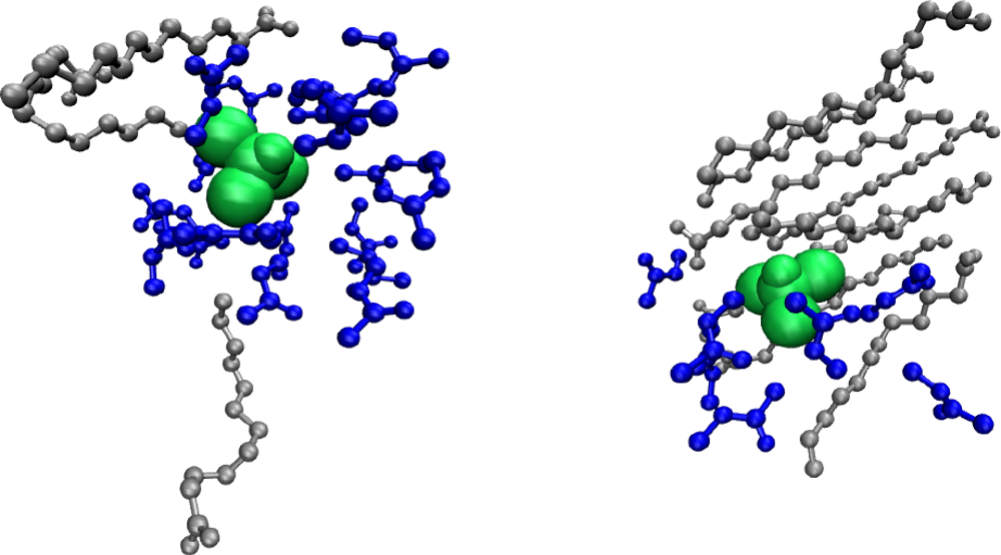
cMD snapshots for CLFd in DES4: on the left the CLFd (green) is found in the NMA domain (blue) interacting with diffused LA molecules (gray), and on the right the CLFd is found along with NMA molecules in open channels of the nonpolar domain. The hydrogen atoms were removed from the structures to enhance visibility.

The DES4 solvation environments predicted for CLFd were compared with experimental results (Supporting Information). To this end, the experimental and theoretical solvatochromism of the C–D stretch of CLFd is investigated in three different molecular solvents (dimethyl sulfoxide, DMSO, tetrahydrofuran, THF, and hexane, HEX). The computations predict a solvatochromism of ∼45 cm^–1^ for the C–D stretch of CLFd in the molecular solvents, which is in good agreement with the experimental value of 33 cm^–1^ (see Supporting Information). Moreover, a strong correlation (*R* = 0.999) is found between the simulation and the experimental results (Figure 9). These results reinforce the idea that the C–D stretch of CFLd is a good probe of the environment and also the applicability of the IFM method for computing the central frequencies of CLFd from cMD simulations.^64^

**Figure 9:**
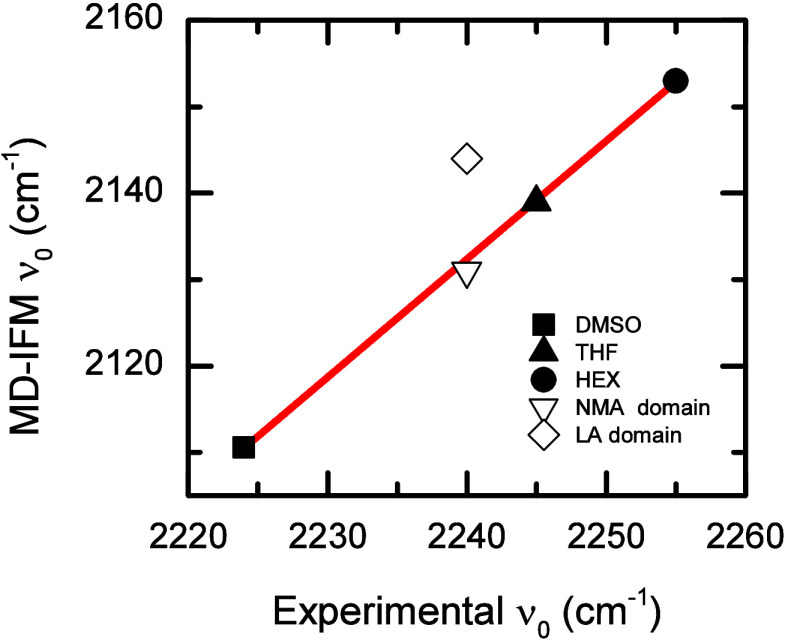
Vibrational solvatochromic shift for the C–D stretch of CLFd in pure solvents and NMA and LA domains of DES4.

As previously shown, the probe molecule is mostly found in the polar domains of the DES4, but it is also found residing within the nonpolar domain. Hence, the IFM method is used to determine the location of the probe. To this end, the central frequency of the C–D stretch of the probe is computed when CLFd is located in either the polar or nonpolar domains of DES4 (see Methods), since the molecular probe can be found in either domain. The result shows that the central frequencies for the probe are either 2131 cm^–1^ when located in the polar domain (NMA) and 2240 cm^–1^ when found in the nonpolar section (LA tails). Using the experimental frequency of 2240 cm^–1^ for the C–D stretch of the probe in DES4 and the linear solvatochromism model derived from the molecular solvents (see Supporting Information), a central frequency of 2132 cm^–1^ is obtained. This value is in excellent agreement with the probe being located mostly within the polar region (Figure 9) and confirms the idea of a CLFd solvation shell formed by both NMA and LA, but mainly NMA, as previously described by using only cMD simulation of the probe (Figure 5b and Supporting Information). Finally, the molecular probe also shows that nonpolar or low polarity molecules can form channels within the LA domains in which they reside, but with low probability due to the large degree of organization and packing of these nonpolar domains.

## Summary

The DESs formed by LA-NMA in 1:4 and 1:6 molar ratios are successfully modeled by cMD simulations. The cMD model shows a heterogeneous molecular structure with a highly ordered nonpolar domain formed by densely packed LA molecules in an interdigitated arrangement. This heterogeneous arrangement of the LA molecules in a matrix of NMA explains the prepeaks in the SAXS structural factors of DES4 and DES6. Moreover, the cMD model shows that the LA domains arise from LA tail–tail interactions, while the NMA domains are formed by single hydrogen bonds between NMA molecules and resemble the structure of pure liquid NMA. Overall, the cMD model suggests that the LA-NMA DES is well described as nanodomains of crystalline-like LA molecules suspended in a liquid matrix of NMA. The cMD model is also tested with a relatively low polarity vibrational probe. The vibrational solvatochromism for the probe in DES4 shows experimentally and computationally that the probe is mainly residing in the NMA polar domain since the tight packing of the LA chains does not allow its insertion into these nonpolar domains.

## Supplementary Material



## Notes

Details on experimental methods, supporting figures and tables containing the evolution of the heterogeneity order parameter for DES4 and DES6, radial distribution functions for the carbon atom of CLFd and the nitrogen atom of NMA and the carboxylic oxygen atom of LA and NMA, distributions of tail to head distances of LA in DES4 and DES6, W­(CO)_6_ molecgular structure, radial distribution functions of the carbon atom of CLFd and the nitrogen atom of NMA or the carboxylic oxygen atom of LA, FTIR spectra of the C–D stretch of CLFd in THF, DMSO, DES4, and HEX, and the linear model for the solvatochromism of CLFd in different solvents (PDF)
