# Supplementary material for: Unraveling the Heterogeneous but Ordered Microstructure of the Nonionic Deep Eutectic Solvent Formed by Lauric Acid and N‑Methylacetamide
Source: J Phys Chem B. 2025 May 30;129(23):5769–78. doi: 10.1021/acs.jpcb.5c00282 (PMC12169661; doi:10.1021/acs.jpcb.5c00282)
Supplement: Supplementary file 1 [file jp5c00282_si_001.pdf]

Supporting information

Unraveling the Heterogeneous but Ordered Microstructure of the Non-Ionic Deep Eutectic Solvent Formed by Lauric Acid and N-Methylacetamide

Laura X. Sepulveda-Montaño,[a] Johan F. Galindo,[b] and Daniel G. Kuroda *,[a]

[a] Department of Chemistry, Louisiana State University, Baton Rouge, Louisiana 70803, United States.

[b] Department of Chemistry, Universidad Nacional de Colombia sede Bogotá, 111321 Bogotá, Colombia.

*Address correspondence to dkuroda@lsu.edu.

FTIR spectroscopy. The linear infrared (IR) spectroscopy measurements were taken in a Bruker tensor 27 FTIR spectrometer equipped with a Nitrogen cooled narrow band MCT detector with a spectral resolution was 0.5 cm^{-1}. The spectra are the result of an average of over 40 scans for all sample except CLFd in LA and hexane which where averaged over 200 scans, to increase the signal to noise ratio. Samples were prepared at ~200mm concentrations of the CLFd in different solvents: Dimethyl sulfoxide (DMSO), Tetrahydrofuran (THF), NMA, and DES4, whereas CLFd in LA and Hex had a concentration of 1M. The samples were placed between CaF2 windows, an optical length of 100μm given by Teflon spacers was used for all measurements. The FTIR measurements above room temperature, for the CLFd in LA and NMA samples, were carried out using a temperature controlled sample cell with a temperature of 45 and 25 ℃, respectively.

Figures

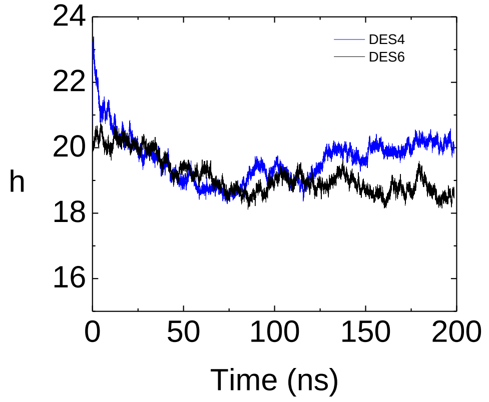

Figure S1.heterogeneity order parameter h for LA in DES4 (red) and DES6 (black) calculated from a cMD simulation of 200ns.

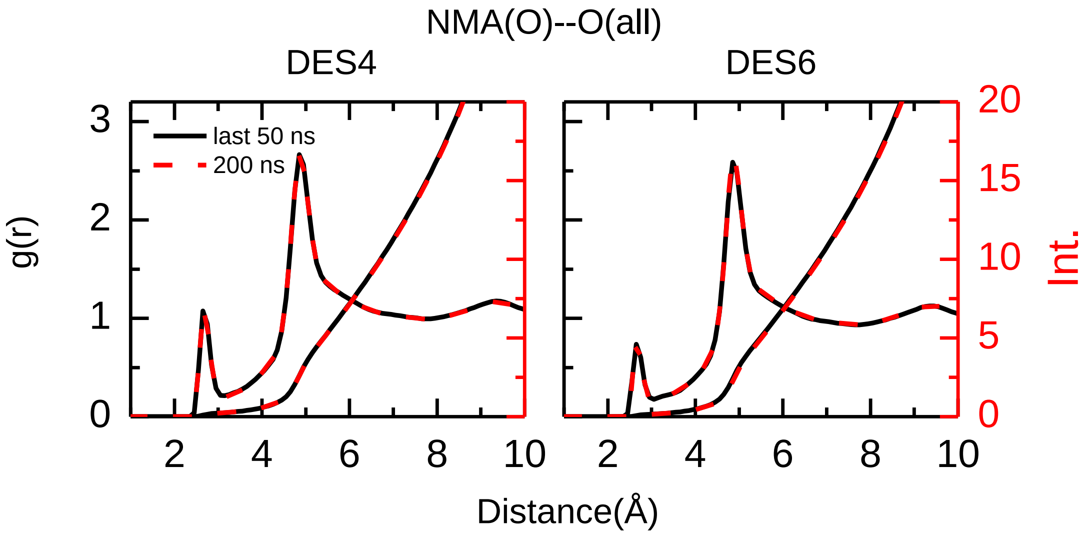

Figure S2. Radial distribution functions (RDF, black left axes) and integrated RDF (red right axes) for the NMA oxygen atom versus all other oxygen atoms in the (a) DES4 and (b) DES6 systems. The black solid and red dashed lines correspond to the RDF calculated from the last 50 ns and the total 200 ns of the MD trajectories, respectively.

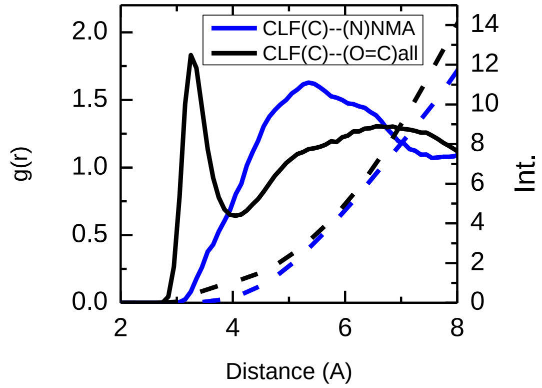

Figure S3. The right axis corresponds to the radial distribution function of the C atom of CLFd respect the N atom of NMA (blue) and the carboxylic O atom of LA and NMA (black). The left axis contains the respective integrals in the same-colored dashed lines.

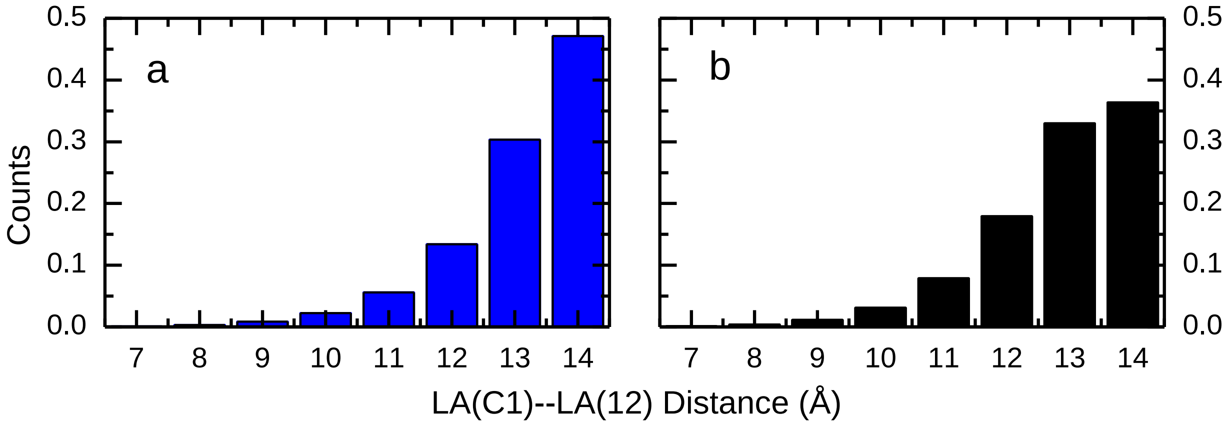

Figure S4. Distribution of distances from C1 to C12 of LA in (a) DES4 and (b) DES6.

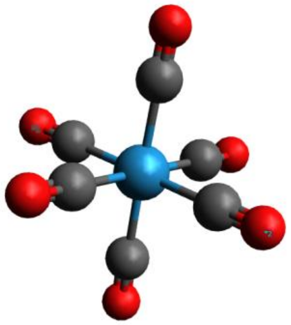

Figure S5. $W(CO)_6$ structure optimized by UFF1 in the Avogadro software,2 the O(1)—O(2) distance measured by this approximate structure corresponds to 6.504Å.

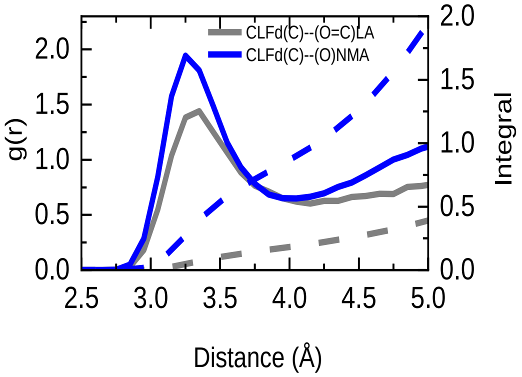

Figure S6. Shows the HB interactions between CLFd and LA and NMA molecules. The right axis corresponds to the radial distribution function of the C atom of CLFd with respect to the N atom of NMA (blue) and the carbonyl O atom of LA (gray). The left axis contains the corresponding integrals in the same colored dashed lines.

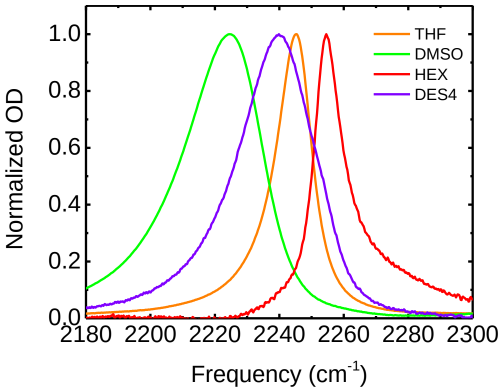

Figure S7. FTIR spectra of the C-D stretch of CLFd in THF (orange), DMSO (green), DES4 (purple), and HEX (red).

$$v_{CD} = 1.37 \pm 0.01 * x - 930 \pm 25$$

Equation 1. Linear model for the solvatochromism of CLFd in different solvents derived from Figure 8 of the main manuscript.

References

1. A. K. Rappé, C. J. Casewit, K. Colwell, W. A. Goddard III and W. M. Skiff, UFF, a full periodic table force field for molecular mechanics and molecular dynamics simulations, *Journal of the American chemical society*, 1992, **114**, 10024-10035.
2. M. D. Hanwell, D. E. Curtis, D. C. Lonie, T. Vandermeersch, E. Zurek and G. R. Hutchison, Avogadro: an advanced semantic chemical editor, visualization, and analysis platform, *Journal of cheminformatics*, 2012, **4**, 1-17.
